# Targeting Melanoma Metastasis and Immunosuppression with a New Mode of Melanoma Inhibitory Activity (MIA) Protein Inhibition

**DOI:** 10.1371/journal.pone.0037941

**Published:** 2012-05-29

**Authors:** Jennifer Schmidt, Alexander Riechers, Raphael Stoll, Thomas Amann, Florian Fink, Thilo Spruss, Wolfram Gronwald, Burkhard König, Claus Hellerbrand, Anja Katrin Bosserhoff

**Affiliations:** 1 Institute of Pathology, University of Regensburg, Regensburg, Germany; 2 Institute of Organic Chemistry, University of Regensburg, Regensburg, Germany; 3 Faculty of Chemistry and Biochemistry, Biomolecular NMR, Ruhr University of Bochum, Bochum, Germany; 4 Department of Internal Medicine I, University Hospital Regensburg, Regensburg, Germany; 5 Institute of Functional Genomics, University of Regensburg, Regensburg, Germany; 6 Institute of Pharmacy, University of Regensburg, Regensburg, Germany; University of Tennessee, United States of America

## Abstract

Melanoma is the most aggressive form of skin cancer, with fast progression and early dissemination mediated by the melanoma inhibitory activity (MIA) protein. Here, we discovered that dimerization of MIA is required for functional activity through mutagenesis of MIA which showed the correlation between dimerization and functional activity. We subsequently identified the dodecapeptide AR71, which prevents MIA dimerization and thereby acts as a MIA inhibitor. Two-dimensional nuclear magnetic resonance (NMR) spectroscopy demonstrated the binding of AR71 to the MIA dimerization domain, in agreement with *in vitro* and *in vivo* data revealing reduced cell migration, reduced formation of metastases and increased immune response after AR71 treatment. We believe AR71 is a lead structure for MIA inhibitors. More generally, inhibiting MIA dimerization is a novel therapeutic concept in melanoma therapy.

## Introduction

MIA, an 11-kDa protein, was identified as strongly expressed and secreted by melanocytic tumor cells, but not by benign melanocytes [1]. MIA expression by melanoma cells correlates strongly with a highly invasive phenotype and the ability to metastasize [2], [3]. Functionally, MIA binds to both extracellular matrix proteins such as fibronectin, laminin and tenascin as well as to specific integrins, cell surface proteins mediating cellular attachment, and thereby contributes to tumor cell detachment and invasion. MIA currently serves as a reliable clinical serum tumor marker for the detection of metastatic diseases and for monitoring responses to therapy [4]. A commercially available MIA-ELISA is routinely used in the follow-up of melanoma patients. Elevated serum levels of MIA correlate with metastatic recurrence and poor prognosis.

The transport of MIA to the cell surface and subsequent secretion is induced after migratory stimuli [5]. MIA then binds to the cell adhesion receptors integrin α_4_β_1_ and integrin α_5_β_1_, which enables tumor cells to invade healthy tissue, resulting in enhanced metastatic potential [6]. In addition to supporting metastatic spread, MIA has also been demonstrated to modulate immunosuppression. This effect is mediated by binding of MIA to integrin α_4_β_1_ expressed by leukocytes [7].

The three-dimensional structure of MIA revealed that MIA defines a novel type of secreted protein with an SH3-domain-like fold [8]. Furthermore, the MIA homologues MIA2 and TANGO have been found to share domains with a high sequence similarity to MIA [9].

## Results and Discussion

**Figure 1:**
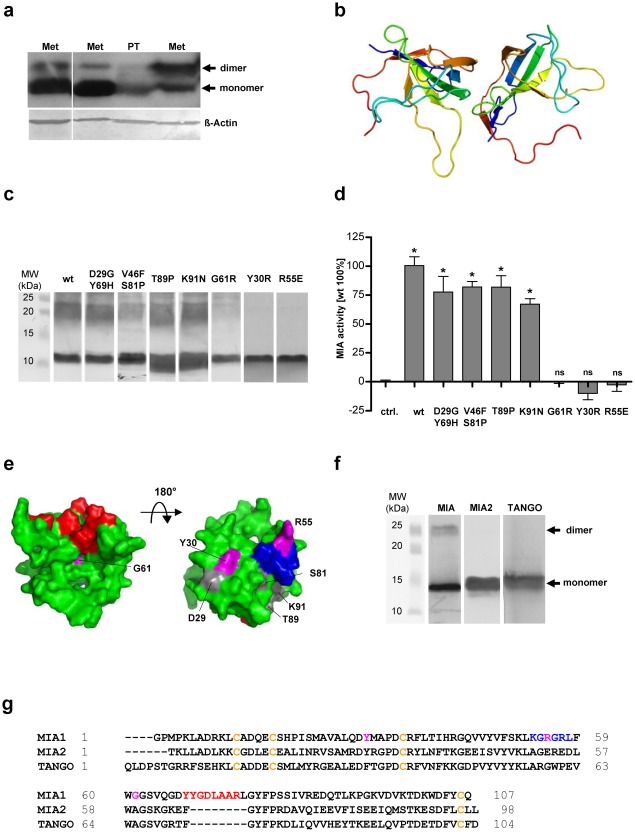
MIA is functionally active as a dimer. (**a**) Western blot analysis of MIA in lysates from melanoma tissue (PT: primary tumor; Met: metastasis) under denaturating conditions. (**b**) The structure of the MIA dimer according to shape complementarity analyses. The MIA dimer is characterized by a head-to-tail orientation, with the dimerization domains consisting of the n-Src loop and the cleft next to the distal loop. (**c**) Western blot analysis of MIA mutants assessing their ability to form dimers. The first lane shows wt MIA, followed by the D29G/Y69H, V46F/S81P, T89P, K91N, G61R, Y30R and R55E mutants. All proteins except for G61R, Y30R and R55E have a clear dimer band. All proteins were electrophoretically resolved from an RTS expression system by SDS-PAGE. (**d**) The correlation between dimerization and functional activity revealed that all MIA mutants capable of dimerization are functionally active in Boyden chamber invasion assays. The G61R, Y30R and R55E mutants which do not form protein dimers, displayed no MIA-induced effect. (**e**) NMR structure of MIA showing the dimerization domains and the mutation sites. The dimerization domains in the n-Src loop and next to the distal loop are depicted in blue and red, respectively. The mutation sites that did not influence dimerization and functional activity are shown in gray. Residues Y30, R55 and G61 are shown in magenta. This figure was generated using PyMol [27]. (**f**) Western blot analysis of MIA and the MIA-homologous MIA2 and TANGO. Only MIA revealed dimerization. (**g**) Sequence alignment of MIA, the N-terminal part of MIA2 and TANGO. Conserved cysteines are shown in yellow. Residues important for MIA dimerization are shown in blue and red. The Y30, R55 and G61 mutation sites are shown in magenta (ns: not significant, *: p<0.05).

Previously, MIA was thought to act as a monomer; however, Western blot analysis of melanoma tissue derived from a primary tumor (PT) or metastases (Met) indicated that stable dimeric species also exist in denaturating SDS-PAGE (**Fig** **1a**). This dimerization is obviously caused by a strong noncovalent interaction since all four cysteins are bound in disulfide bridges intramolecularly [8] which excludes the possibility of intermolecular disulfide bridges. Using PreBI modeling software (http://pre-s.protein.osaka-u.ac.jp/prebi/) to predict the putative dimer interface and the HADDOCK protein-protein docking program [10], we obtained a model of the MIA dimer that included a head-to-tail linkage (**Fig** **1b**). The dimerization interfaces are located around the K53-L58 region in the n-Src loop and the cleft next to Q65-A73 in the distal loop, as defined by the MIA 3D structure [8]. The amino acid residues Y30, R55 and G61 were predicted by these *in silico* studies to be particularly important for dimerization. Interestingly, the same regions that we determined to form the interfaces were described as crucial for MIA activity in a previous mutagenesis study [11]. We therefore investigated the possible correlation between MIA dimerization and functional activity. Having identified the most likely positions of the dimerization interfaces, different mutants of MIA (D29G/Y69H, V46F/S81P, T89P, K91N, G61R, Y30R and R55E) were tested for their ability to form dimers by Western blot analysis (**Fig** **1c**). Wild-type (wt) MIA and all mutants except for G61R, Y30R and R55E clearly showed a dimer band. As predicted, the mutations affected the putative dimerization domains.

To correlate the functional activity of the different MIA mutants with dimerization, Boyden chamber invasion assays were performed with Mel-Im melanoma cells (**Fig** **1d**). All mutations preventing dimerization (Y30R, R55E, G61R) also led to a loss of activity. The sites of the mutations that did not affect functional MIA activity (**Fig** **1e**, depicted in gray) are located outside the dimerization regions, whereas Y30, R55 and G61 (**Fig** **1e**, depicted in magenta) are located in proximity to the dimerization domains (red and blue). These results indicate that the MIA binding site for extracellular matrix structures and integrins is only formed upon dimerization. Interestingly, the amino acids required for dimerization are highly conserved (**Fig** **S1**).

We further analyzed the MIA-homologous proteins MIA2 and TANGO, two members of the MIA protein family [12]. Western blotting demonstrated that these MIA homologues do not form dimers (**Fig** **1f**), which is in agreement with the sequence alignment demonstrating that the amino acids crucial for dimerization are not conserved in MIA2 and TANGO (**Fig** **1g**). Furthermore, the MIA dimerization domain in the n-Src loop (K53-L58) shows an inversion of charge in MIA2 and TANGO. The dimerization domain in the distal loop shows a large deletion in MIA2 and TANGO, with Y68-R75 being absent in both of these MIA homologues (**Fig** **1g**).

**Figure 2:**
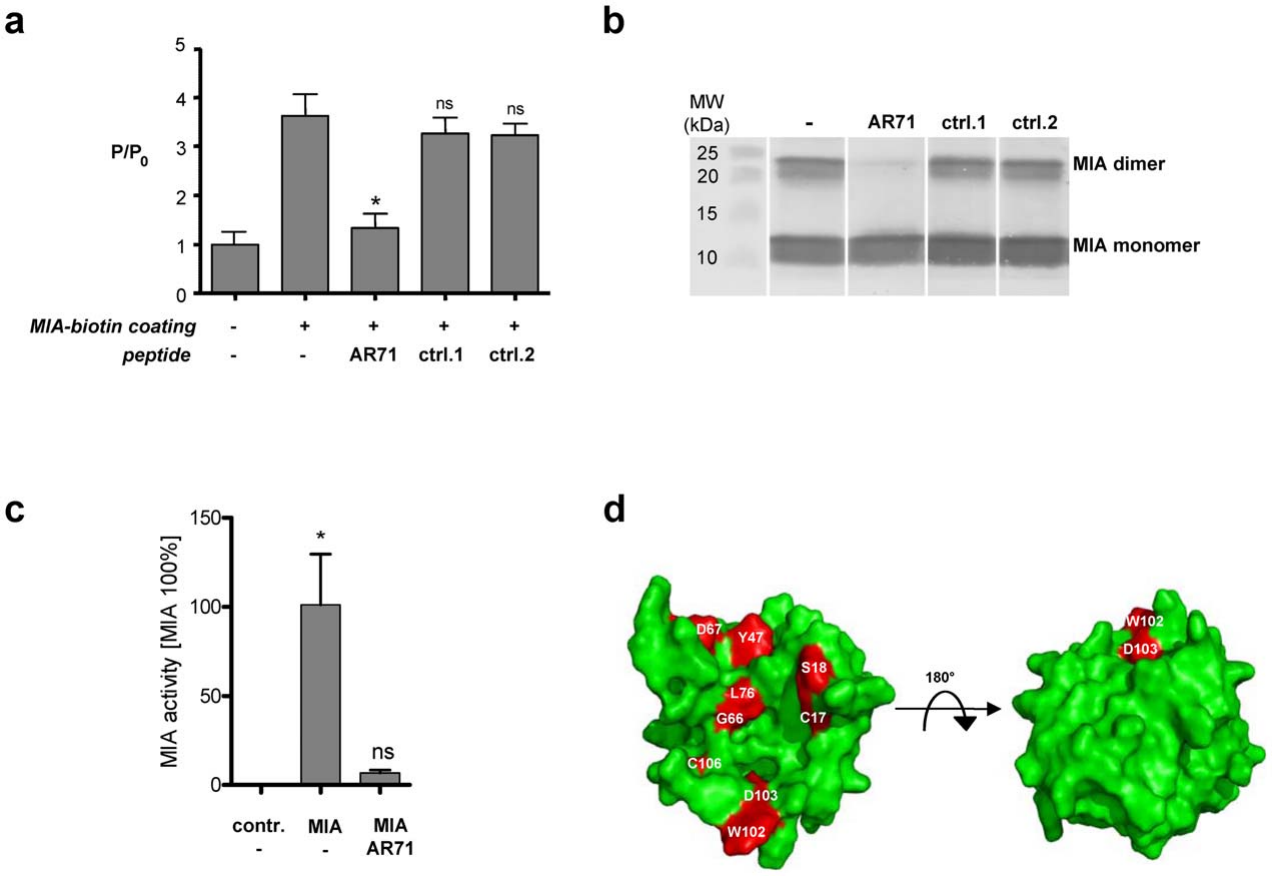
Peptide AR71 prevents MIA dimerization. (**a**) The HTFP assay was used to assess the ability of AR71 to directly interfere with the MIA-MIA interaction. Fluorescence polarization was normalized (P/P_0_) to the fluorescence polarization of MIA-Ru(bpy)_3_ in a well not coated with MIA-biotin. First, the FP signal of MIA-Ru(bpy)_3_ was measured in a well that was coated with MIA-biotin and compared to the signal in an uncoated well. The significant increase in FP in the well coated with MIA-biotin indicates binding of MIA-Ru(bpy)_3_ to the immobilized MIA-biotin. The binding of AR71 (1.6 µM) displaced the surface-bound MIA-Ru(bpy)_3_, as reflected by a decrease in the fluorescence polarization signal. MIA-binding control peptides 1 and 2 (1.6 µM) did not interfere with the MIA-MIA interaction. (**b**) Western blot analysis of MIA incubated with AR71 demonstrated peptide-induced dissociation of the dimer. Control peptides 1 and 2 did not lead to reduced dimer formation. (**c**) Boyden chamber invasion assays using Mel-Im cells indicated that AR71 almost completely inhibited MIA activity. Pre-incubation of MIA with AR71 resulted in neutralization of the MIA effect, while AR71 alone did not influence migratory behavior. The final concentrations were 200 ng/mL for MIA and 1 µM for AR71. (**d**) The most significant chemical shift differences projected onto the van der Waals surface of MIA upon titration with AR71 are depicted in red. The binding site is located in the dimerization domain next to the distal loop (compare to **Figure** **1e**). This figure was generated using PyMol [27]. (ns: not significant, *: p<0.05).

To identify peptides inhibiting MIA dimerization, we applied a newly developed heterogeneous transition-metal-based fluorescence polarization (HTFP) assay [13]. First, the MIA-MIA interaction was confirmed using this assay. We immobilized a MIA-biotin conjugate in a streptavidin-coated well plate and added MIA labeled with the luminescent transition-metal complex Ru(bpy)_3_. As depicted in **Figure** **2a**, a significant increase in the fluorescence polarization (FP) signal was observed in the wells coated with MIA-biotin compared to control wells. This increase was attributed to the severely restricted rotational mobility of MIA-Ru(bpy)_3_ bound to the immobilized MIA-biotin. We then screened peptides, previously identified by phage display and known to generally bind to MIA [8], for their potential to prevent MIA dimerization and to induce dissociation of already existing protein dimers using the HTFP assay. The dodecapeptide AR71 (sequence: Ac-FHWRYPLPLPGQ-NH_2_) effectively dissociated MIA dimers, which led to a decrease in FP due to increased rotational diffusion of the dissociated monomeric MIA-Ru(bpy)_3_, while other dodecapeptides (control peptides 1, sequence: Ac-VSNYKFYSTTSS-NH_2_ and 2, sequence: Ac-YNLPKVSSNLSP-NH_2_) did not affect the FP signal.

The inhibitory effect of AR71 on MIA dimerization was confirmed by Western blot analysis (**Fig** **2b**). Preincubation of MIA with AR71 led to a strong reduction of the dimer band compared to MIA without peptides or with MIA binding control peptides 1 and 2. This inhibition of dimerization was also observed in native PAGE experiments (**Fig** **S2**).

Boyden chamber invasion assays revealed that AR71 also functionally inhibits MIA (**Fig** **2c**). Preincubation of MIA with AR71 resulted in complete neutralization of the effect of MIA on melanoma cells. The results were confirmed in three other melanoma cell lines (Mel-Ju, Mel-Wei, and Mel-Ho; data not shown). We further tested AR71 in classical migration assays. Significant inhibition of melanoma cell migration was observed using two cell lines in three independent assays (**Fig** **S1b**). These assays also indicated that AR71 does not induce apoptosis *in vitro*.

In line with the inhibitory effect of AR71 on the formation of MIA aggregates, the direct binding of AR71 to the MIA dimerization domain next to the distal loop was shown by multidimensional NMR spectroscopy. By using increasing amounts of AR71, the induced chemical shift changes of the MIA ^1^H^N^ and ^15^N^H^ resonances were classified according to the degree of the combined chemical shift perturbations. Further analysis of the solvent accessibility (with a threshold of 20%) and cluster analysis of the residues affected by peptide binding revealed that the binding interface comprises residues C17, S18, Y47, G66, D67, L76, W102, D103 and C106 in MIA (**Fig** **2d**). These data indicate that the peptide binds to the site depicted in red in **Figure** **2d**, whereas the opposite side of the molecule does not participate in AR71 binding. The AR71 binding site is located in the cleft next to the distal loop, where the G61R mutation leading to functional inactivation is situated (**Fig** **1e**). HTFP and Western blot analyses confirmed the ability of AR71 to monomerize MIA (**Fig** **2a and 2b**). The concave nature of the AR71 binding site makes it especially attractive as a druggable target due to its large surface area and suitable geometry for binding small-molecule pharmacophores that could be derived from AR71.

Next, we analyzed the interaction of AR71 with endogenous MIA in B16 mouse melanoma cells stably transfected with a secretion signal containing the AR71-HisTag construct (AR71-HisTag). With the addition of an N-terminal secretion sequence ensuring peptide processing in the endoplasmic reticulum, we expected subsequent binding and thus inactivation of MIA directly at the location of protein biosynthesis.

**Figure 3:**
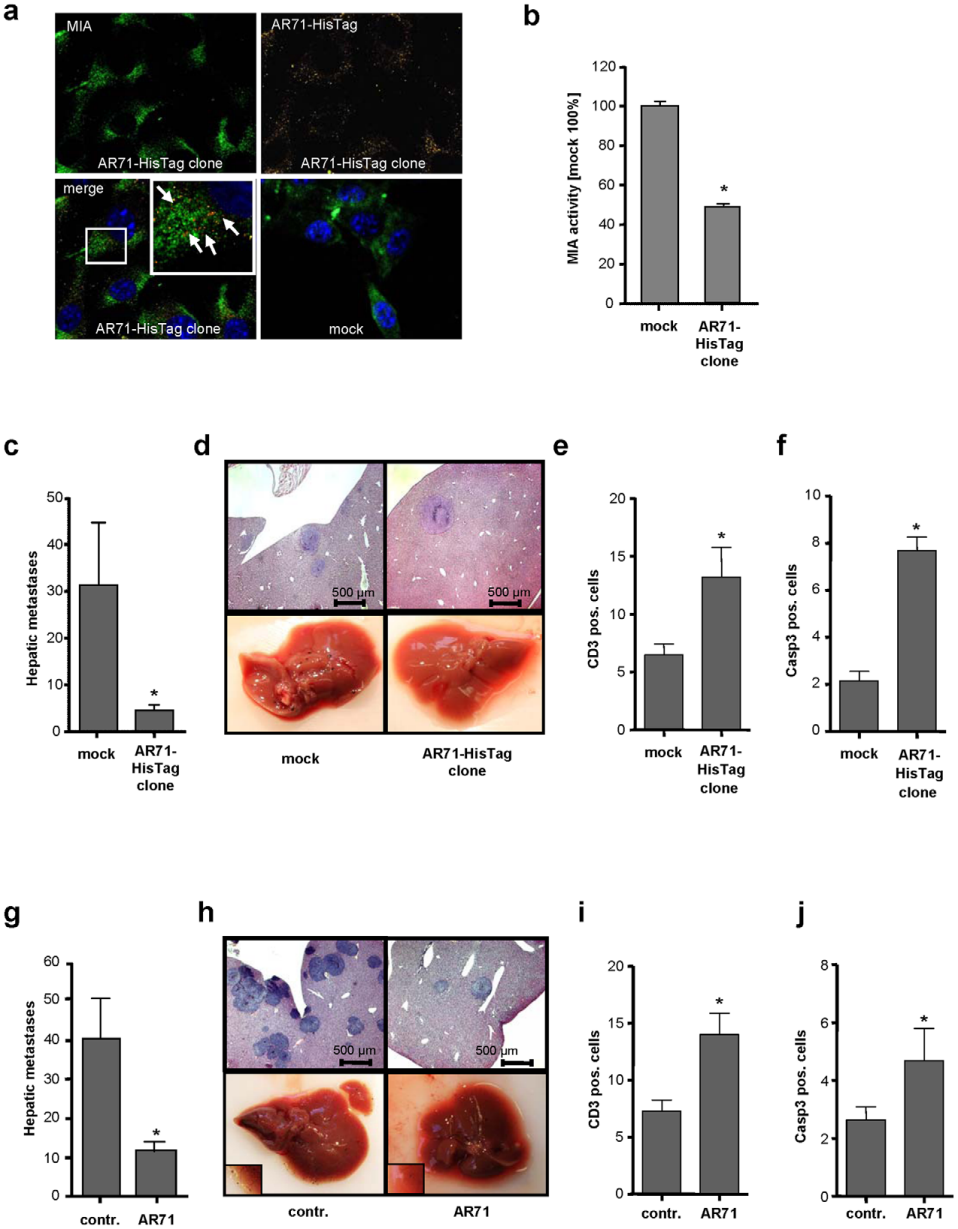
Effect of MIA inhibitory peptide AR71 on the formation of metastases *in vivo*. (**a**) Immunofluorescence studies of murine B16 melanoma cells stably transfected with an AR71-HisTag construct, with MIA (FITC) shown in green and AR71-HisTag (TRITC) shown in yellow. Co-localization is depicted in red and is indicated by white arrows. The excess of MIA not co-localized with AR71 is due to the internalization of exogenous MIA by the melanoma cells [29]. The mock control did not include AR71-HisTag. (**b**) Murine B16 AR71-HisTag clones were analyzed for their migratory activity in a Boyden chamber assay. Compared to the mock control, the AR71-HisTag-expressing cell clone displayed drastically reduced migration by reducing MIA activity. (**c**) The AR71-HisTag clone and a corresponding mock control were injected into the spleens of Bl/6N mice (n = 8 each). Histological analysis revealed that AR71-HisTag clones formed significantly fewer hepatic metastases than the mock control cells did. (**d**) Representative histological liver sections (upper row) and macroscopic pictures (lower row) of mice injected with the mock control and an AR71-HisTag-expressing cell clone, respectively. (**e**) Anti-CD3 immunohistochemistry revealed an increase in the number of T-lymphocytes in the livers of mice that received AR71-HisTag-expressing cells. (**f**) Anti-caspase 3 immunohistochemistry indicated increased caspase 3-mediated apoptosis in the AR71-HisTag-expressing tumors. (**g**) Wild-type murine B16 melanoma cells were injected into the spleens of Bl/6N mice before treatment with *i.v.* injections of AR71 (n = 8 each). Histological analyses revealed a significant reduction in the average number of hepatic metastases in mice treated with AR71 as compared to untreated control mice. (**h**) Representative histological liver sections (upper row) and macroscopic pictures (lower row, magnifications in lower left corners) of untreated (control) and treated (AR71) mice. (**i**) Anti-CD3 immunohistochemistry revealed an increase in the number of T-lymphocytes in the livers of mice treated with AR71. (**j**) Anti-caspase 3 immunohistochemistry indicated increased caspase 3-mediated apoptosis in the tumors of mice treated with AR71. (*: p<0.05).

We first investigated the expression and localization of the AR71-HisTag peptide by immunofluorescence analysis. Co-staining of MIA and AR71-HisTag revealed co-localization in close proximity to the nucleus (**Fig** **3a**).

While no effect of AR71-HisTag on cell growth was observed, functional *in vitro* analysis confirmed that migration is drastically reduced in AR71-HisTag-expressing cell clones compared to mock-transfected control cells (**Fig** **3b**), and HTFP analysis demonstrated that AR71-HisTag interferes with the MIA-MIA interaction (data not shown).

Because *in vivo* studies have demonstrated the strong contribution of MIA to melanoma cell invasion and migration [14], [15] we aimed to assess the effect of AR71 on the metastasis of melanoma cells and on the immune response *in vivo*. Therefore, we employed an established syngeneic murine model of hepatic metastasis [16] using the AR71-HisTag-containing cell clones. Notably, mice injected with AR71-HisTag melanoma cell clones had significantly fewer hepatic metastases than mice that received mock-transfected control cells (**Fig** **3c and 3d**).

Additionally, immunohistochemistry analysis revealed an increase in the number of CD3^+^ cells and in the level of caspase 3-induced apoptosis in the hepatic metastases of mice that received the AR71-HisTag clone (**Fig** **3e and 3f**, **Fig** **S3a** and **S3b**), indicating an inhibition of MIA-induced immunosuppression by AR71.

These results prompted us to investigate whether the formation of metastases could also be inhibited by intravenous administration of AR71. In this experimental setting, wild-type B16 melanoma cells were used in the hepatic metastasis model, and AR71 (2.5 mg/kg every 24 h) or solvent control was administered intravenously for 9 days. Histological analysis revealed a significant reduction in the formation of hepatic metastases upon treatment with AR71 (**Fig** **3g and 3h**). Increased numbers of CD3^+^ cells and activated caspase 3 were detected in the hepatic metastases of mice treated with AR71 (**Fig** **3i and 3j**, **Fig** **S4a** and **S4b**).

No adverse effects were observed (data not shown). Although peptides are generally degraded quickly *in vivo* by proteases and are cleared renally, this study demonstrated the potency of AR71 in suppressing the metastatic spread and immunosuppression of melanoma cells *in vivo*.

This study details a two-pronged approach of targeting both the metastatic spread and immunosuppression of melanoma and provides a novel leading structure for the design of potent therapeutics for the treatment of melanoma. Most conventional treatments still affect fast-dividing cell types in addition to the cancer cells. There is an urgent need for better targeted therapies. By targeting MIA, which is only expressed in melanoma and in differentiating chondrocytes, the adverse reactions of treatment with MIA inhibitory compounds should be minimal. Side effects on cartilage are not expected because MIA-deficient mice show only minor phenotypic changes [17]. Currently used targeted melanoma treatment regimes include Vemurafenib which targets the BRAF V600E mutation and Imatinib which targets activating c-Kit mutations. Conceptually, a MIA inhibitor would be beneficial for all melanoma patients since it is not dependent on mutations which are only present in a subpopulation of patients. Furthermore, we envision that a MIA inhibitor could be given in combination with other treatments since it targets a novel pathway of melanoma progression.

We believe that this study provides an excellent starting point for the development of a new strategy in melanoma therapy. Targeting MIA leads to strongly reduced formation of metastases and immunosuppression and thus provides a new concept for therapeutic intervention.

## Materials and Methods

### Cell lines and cell culture conditions

The melanoma cell line Mel-Im, established from a human metastatic bioptic sample (generous gift from Dr. Johnson, University of Munich, Germany) was used in all experiments. Additionally, main experiments were also conducted using the human cell line Mel-Ju and the murine cell line B16, which were derived from metastases of malignant melanoma, as well as Mel-Wei and Mel-Ho cells derived from primary human melanoma. All cells were maintained in DMEM (PAA Laboratories GmbH, Cölbe, Germany) supplemented with penicillin (400 U/mL), streptomycin (50 µg/mL), L-glutamine (300 µg/mL) and 10% fetal calf serum (Pan Biotech GmbH, Aidenbach, Germany) and split in 1∶6 ratio every 3 days.

### In vitro spheroid and scratch assays

Spheroid collagen invasion degradation assays were performed as described previously [18]. Spheroid migration assays were performed as described [19], except that quantification of migration was not performed by manual measurements using a microscope but via the Roche xCELLigence system (Roche, Mannheim, Germany). Scratch migration assays were performed as described previously [20]. In all assays, AR71 peptide was used at a final concentration of 1 µM.

### Protein analysis in vitro (Western blotting)

Protein extraction from tumor tissue and Western blotting were performed as described previously [21], [13]. In the multimerization studies, MIA (1 µg) was incubated with AR71 (2.5 µg) overnight at RT before being subjected to Western blot analysis. Purified antibodies which do not detect other MIA isoforms were used throughout all experiments. MIA mutant expression plasmids were prepared from the wild type MIA-piVEX2.3-MCS plasmid [11] by using the QuikChange II Site-Directed Mutagenesis Kit (Agilent, La Jolla, CA, USA) according to the manufacturer's instructions. MIA mutants were expressed using the cell free Rapid Translation System E. coli HY Disulfide Kit (5Prime, Hamburg, Germany) as described previously [11].

### Boyden Chamber Invasion Assay to determine MIA activity

Invasion assays were performed in Boyden Chambers containing polycarbonate filters with 8-µm pore size (Neuro Probe, Gaithersburg, MD, USA) essentially as de­scribed [11]. MIA was added to the cell suspension at a final concentration of 200 ng/mL. Peptide AR71 (sequence: Ac-FHWRYPLPLPGQ-NH_2_) was used at a final concentration of 1 µM. Experiments were carried out in triplicates and repeated at least three times.

### Polarization assay setup

Black, streptavidin coated 96 well plates (Greiner Bio-one, Frickenhausen, Germany) were coated with MIA-biotin as described and all measurements were performed at RT on a Polarstar Optima microplate reader (BMG Labtech, Offenburg, Germany) as described [13]. Briefly, human MIA protein (100 µg) was labeled with Ru(bpy)_3_-isothiocyanate (1 mg, Active Motif Chromeon, Tegernheim, Germany) and purified over a size exclusion column (Sephadex G75 M PD-10, Amersham Pharmacia Biotech, Uppsala, Sweden). After coating with MIA-biotin, addition of compounds to the well plate was done in the following order: buffer, peptide, MIA-Ru(bpy)_3_. A total volume of 250 µL was used per well. Polarization values are reported relative (P/P_0_) to the value of free MIA-Ru(bpy)_3_ in solution in a well not treated with MIA-biotin. Reported values represent an average of three independent measurements.

### Cloning Strategy

The Signal-AR71-HisTag pCMX-PL1 expression plasmid was created by PCR amplification of the human hydrophobic signal-peptide sequence, responsible for transport into the endoplasmic reticulum, from a Signal-MIA containing expression plasmid. The HisTag sequence was inserted at the C-terminal end of the AR71 peptide using the primers 5′-GACGAATTCATGGCCCGGTCCCTGGTG-3′ and 5′-GACAAGCTTTCAGTGATGGTGATGGTGATGCTGGCCGGGCAAGGGCAAGGGGTATCTCCAGTGGAACCTGACACCAGGTCCGGAGAA-3′. After amplification of the Signal-AR71-HisTag fragment, the PCR product was digested with Eco*RI* and Hind*III* (NEB, Frankfurt, Germany). The insert was purified by gel extraction (Qiagen, Hilden, Germany) and cloned into the Eco*RI* and Hind*III* sites of pCMX-PL1 [22]. The sequence of the PCR-generated clone was confirmed by DNA sequencing.

### Stable transfection of murine B16 melanoma cells

Stable cell lines expressing AR71-HisTag were generated as described [14]. After selection of cells comprising antibiotic resistance we confirmed expression and localization of AR71 peptide on mRNA and protein level by qRT-PCR and immunofluorescence, respectively.

### Recombinant expression of MIA and mutant forms

*In vitro* protein expression reactions of recombinant human MIA and its mutants were performed with the Rapid Translation System RTS 500 E. coli HY Disulfite Kit (Roche, Mannheim, Germany) according to the manufacturer's instructions. MIA mutants were checked for correct folding and function as described [11].

### NMR Spectroscopy

All spectra were recorded at 300 K and pH 7 on a Bruker DRX600 spectrometer equipped with a pulsed field gradient triple resonance probe. Water suppression in experiments recorded on samples in H_2_O was achieved by incorporation of a Watergate sequence into the various pulse sequences [23], [24]. 2D ^1^H-^15^N HSQC spectra with reduced signal loss due to fast exchanging protons were recorded using procedures described previously [25]. All spectra were processed with NMRPipe and analyzed with NMRView [26]. Data handling was performed with NMRView. Structure visualisation and superimpositions were done with the PyMol software [27].

### Dimer model

The PreBI modeling software (http://pre-s.protein.osaka-u.ac.jp/prebi/) was used together with the published X-ray structure of MIA (PDBid: 1I1J) for the prediction of the putative dimer interface. Employing the monomeric NMR structure of MIA (PDBid: 1HJD) together with the interface information obtained in the previous step a three-dimensional model of the dimeric complex was calculated. Computations were performed using the data driven protein-protein docking program HADDOCK [10].

### Protein binding studies

The NMR titration of MIA with AR71 consisted of monitoring changes in chemical shifts and line widths of the backbone amide resonances of uniformly ^15^N-enriched MIA samples as a function of ligand concentration [28].

### In vivo metastasis assay

To determine the effect of peptide AR71 on the metastatic potential of murine B16 melanoma cells *in vivo*, a previously developed mouse metastases model was used [16]. 1×10^5^ cells (in 50 µl) of the AR71-HisTag expressing B16 cell clone AR71-HisTag or the corresponding mock control cells were injected into the spleen of syngeneic Bl/6N mice (n = 8 each). After nine days the mice were sacrificed, the livers were resected and the number of visible black tumor nodules on the surface of the livers was documented. Tissues were fixed in formalin, and afterwards, paraffin embedded sections were hematoxylin and eosin stained for histological analysis.

In a second experimental setting, 1×10^5^ wt mouse melanoma B16 cells suspended in a solution containing AR71 (1 mg/mL) and 0.9% NaCl (or only 0.9% NaCl in the control group) were injected in 50 µl into the spleen of Bl/6N mice (n  = 8 each group). Peptide AR71 was injected *i.v.* (2.5 mg/kg in 50 µL every 24 h). After nine days, the mice were sacrificed and the livers were resected. Also here, formation of tumor nodules was documented. Following fixation with formalin and paraffin embedding, hematoxylin and eosin stained sections for histological analysis were generated.

Immunohistological stainings were performed using routine diagnostic procedures and anti-CD3 (#RM-9107, NeoMarkers, Fremont, California) and anti-Caspase 3 (#9661, Cell Signaling, Frankfurt, Germany) antibodies. For quantification of immunohistological stainings, livers from every mouse per group were evaluated.

### Immunofluorescence assays

Cells were grown in a 4-well chamber slide (BD Bioscience, Heidelberg, Germany) for 48 h and fixed using 4% paraformaldehyde in 0.1 M phosphate-buffered saline (PBS) for 15 min. After permeabilization of cells and blocking of non-specific binding sites (1% BSA/PBS, 1 h, 4°C) cells were incubated with primary antibodies rabbit anti-MIA (Biogenes, Berlin, Germany) and mouse anti-HisTag (BD Bioscience) at a concentration of 1 µg/mL at 4°C for 2 h. After washing steps, secondary antibodies TRITC anti-mouse (1∶200, Jackson Immuno Research Laboratories, West Grove, PA, USA) and FITC anti-rabbit (1∶200, DakoCytomation, Hamburg, Germany) were added. Following incubation with secondary antibodies, cells were washed with PBS and coverslips were mounted on slides using Hard Set Mounting Medium with DAPI (Vectashield, H-1500, Linearis, Wertheim Germany) and imaged using an Axio Imager Zeiss Z1 fluorescence microscope (Axiovision Rel. 4.6.3) equipped with an Axio Cam MR camera. Images were taken using 63x oil immersion lenses.

### Statistical analysis

Results are expressed as mean ± S.E.M. or percent. Comparison between groups was made using the Student's unpaired t-test. A p-value <0.05 was considered as statistically significant (ns: not significant, *: p<0.05). All calculations were made using the GraphPad Prism Software (GraphPad Software, Inc., San Diego, USA).

## Supporting Information

Figure S1 **Species conservation of MIA and** ***in vitro*** **inhibition by AR71.** (**a**) The MIA protein sequence is highly conserved throughout various species. All four cysteines (orange) and Y30 and R55 (green) are completely conserved. G61 (magenta) is mostly conserved; *Salmo salar* and *Danio rerio* have an alanine at position 61; alanine has electrostatic properties similar to those of glycine. (**b**) Additional functional assays were performed (I: collagen invasion assay; II: Scratch Assay, III: Spheroid migration assay) to confirm the inhibitory effects of AR71 on melanoma cell migration and invasion in two melanoma cell lines (Mel-Im, Mel-Ju).
(TIF)

Figure S2 **Native PAGE analysis of MIA incubated with AR71 demonstrated peptide-induced dissociation of the MIA dimer.**
(TIF)

Figure S3 **Immunohistochemical evaluation of murine tissues.** (**a**) Representative CD3-stained liver sections of mice treated with B16 mock or B16 AR71-HisTag cells. (**b**) Representative caspase 3-stained liver sections from mice treated with B16 mock or B16 AR71-HisTag cells. Scale bars are 100 µm.
(TIF)

Figure S4 **Immunohistochemical evaluation of murine tissues.** (**a**) Representative CD3-stained liver sections from solvent control and AR71-treated mice. (**b**) Representative caspase 3-stained liver sections from solvent control and AR71-treated mice. Scale bars are 100 µm.
(TIF)
