# Supplementary figures and images for: Targeting Melanoma Metastasis and Immunosuppression with a New Mode of Melanoma Inhibitory Activity (MIA) Protein Inhibition
Source: PLoS One. 2012 May 29;7(5):e37941. doi: 10.1371/journal.pone.0037941 (PMC3362532; doi:10.1371/journal.pone.0037941)

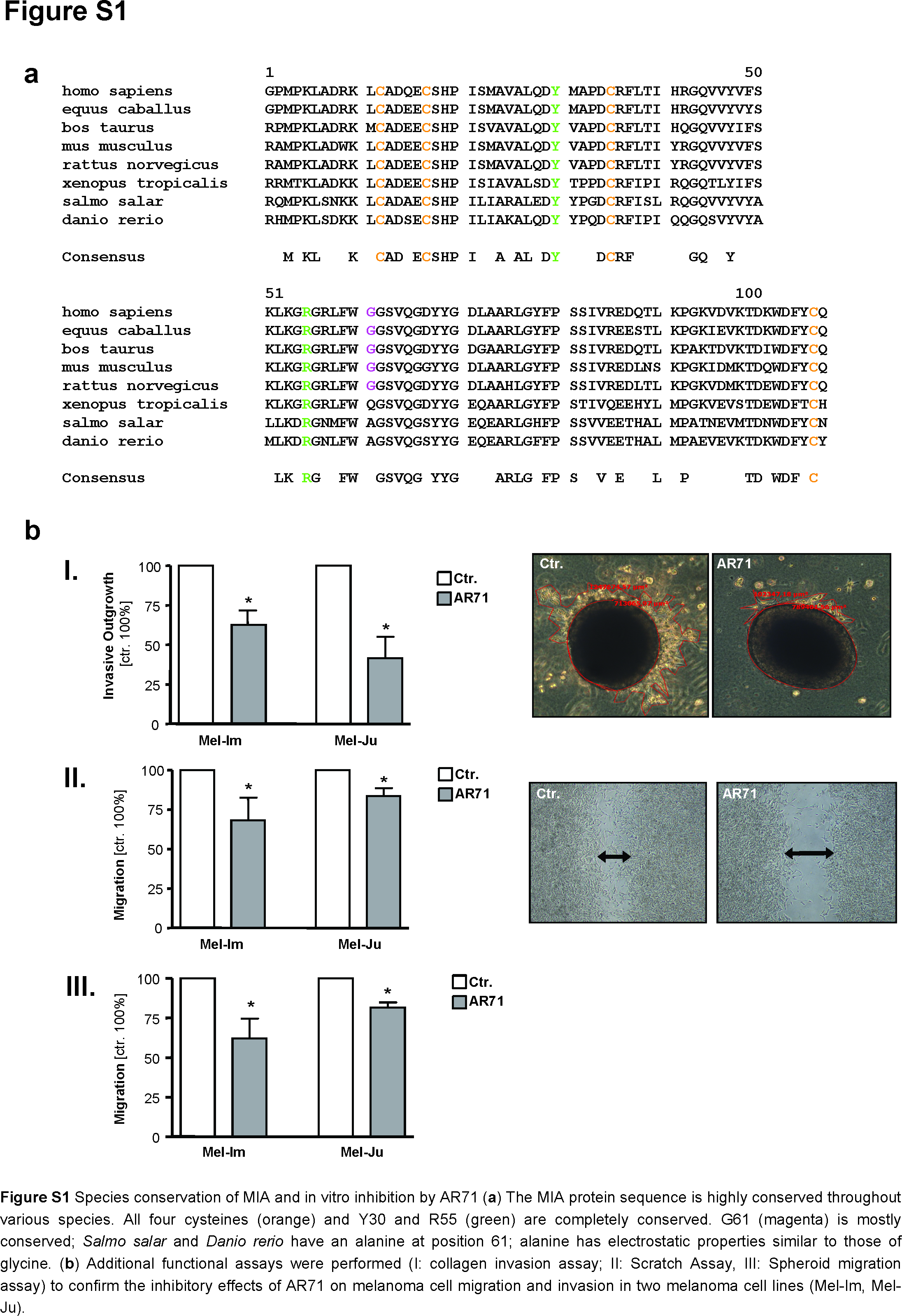

Supplement: Figure S1 — Species conservation of MIA and in vitro inhibition by AR71. (a) The MIA protein sequence is highly conserved throughout various species. All four cysteines (orange) and Y30 and R55 (green) are completely conserved. G61 (magenta) is mostly conserved; Salmo salar and Danio rerio have an alanine at position 61; alanine has electrostatic properties similar to those of glycine. (b) Additional functional assays were performed (I: collagen invasion assay; II: Scratch Assay, III: Spheroid migration assay) to confirm the inhibitory effects of AR71 on melanoma cell migration and invasion in two melanoma cell lines (Mel-Im, Mel-Ju). (TIF) [file pone.0037941.s001.tif]

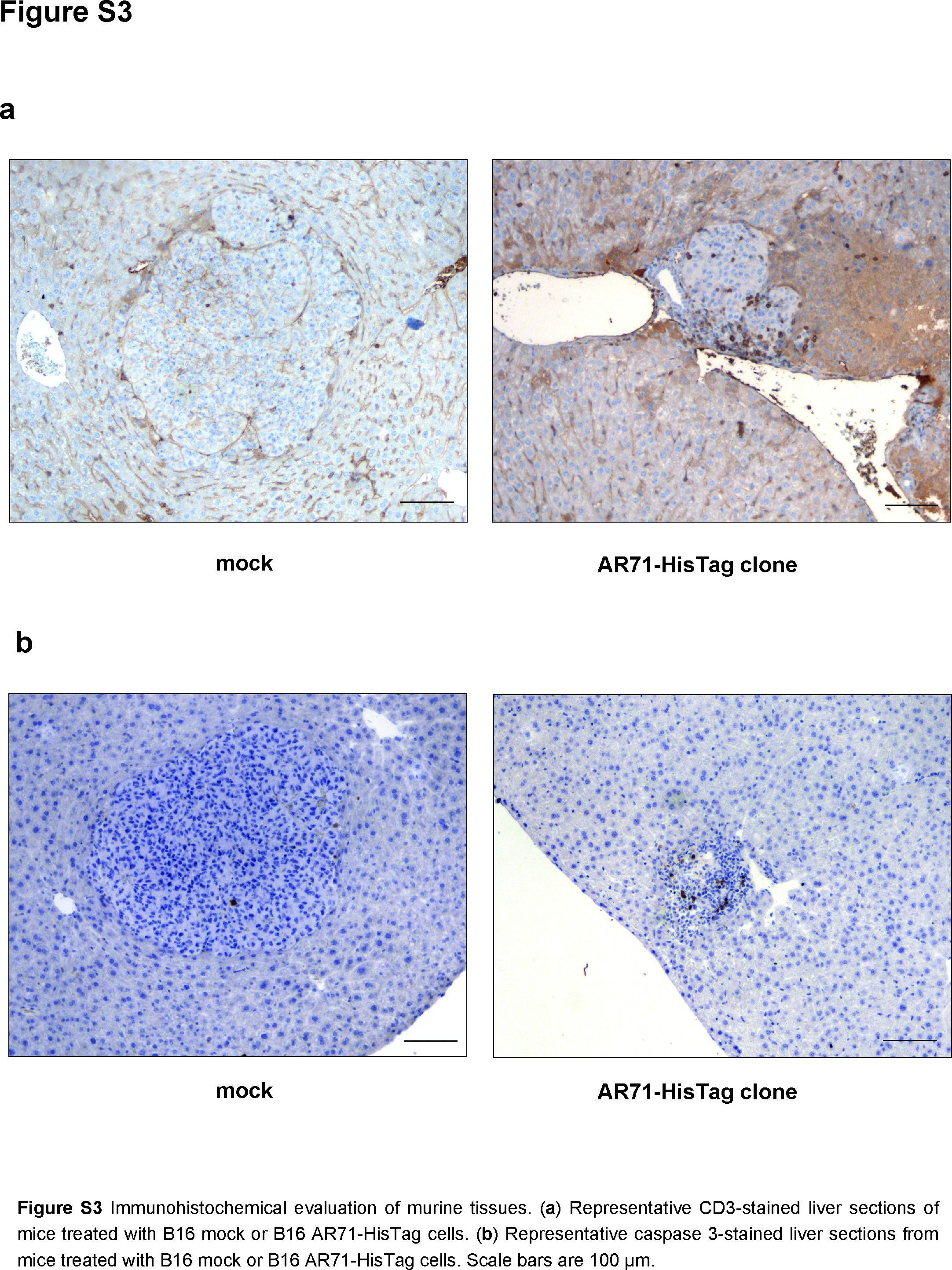

Supplement: Figure S3 — Immunohistochemical evaluation of murine tissues. (a) Representative CD3-stained liver sections of mice treated with B16 mock or B16 AR71-HisTag cells. (b) Representative caspase 3-stained liver sections from mice treated with B16 mock or B16 AR71-HisTag cells. Scale bars are 100 µm. (TIF) [file pone.0037941.s003.tif]

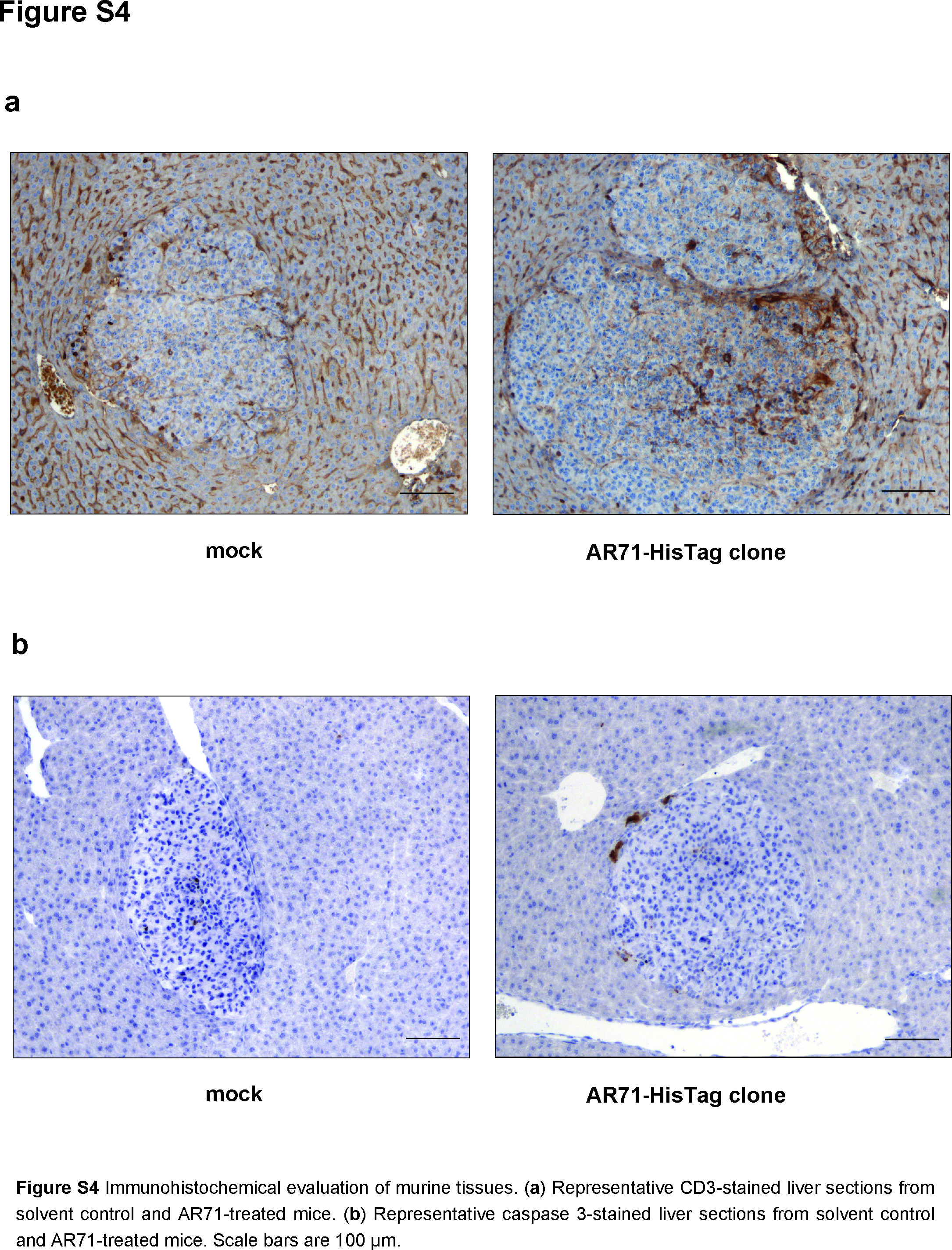

Supplement: Figure S4 — Immunohistochemical evaluation of murine tissues. (a) Representative CD3-stained liver sections from solvent control and AR71-treated mice. (b) Representative caspase 3-stained liver sections from solvent control and AR71-treated mice. Scale bars are 100 µm. (TIF) [file pone.0037941.s004.tif]
